# CCL22 and Leptin associated with steroid resistance in childhood idiopathic nephrotic syndrome

**DOI:** 10.3389/fped.2023.1261034

**Published:** 2023-09-08

**Authors:** Peng Zhaoyang, Li Wei, Jin Yanyan, Xiang Wenqing, Fu Haidong, Mao Jianhua

**Affiliations:** ^1^ Department of Clinical Laboratory, Children’s Hospital, Zhejiang University School of Medicine, National Clinical Research Center for Child Health, Hangzhou, China; ^2^ Department of Nephrology, Children’s Hospital, Zhejiang University School of Medicine, National Clinical Research Center for Child Health, Hangzhou, China

**Keywords:** CCL22, leptin, steroid-resistant nephrotic syndrome, T regulatory cells, idiopathic nephrotic syndrome

## Abstract

**Objective:**

Previous studies have indicated a decrease in T regulatory cells (Tregs) among patients with steroid-resistant nephrotic syndrome. CCL22 and Leptin influenced the immune function of Tregs through their respective pathways. This study aimed to compare patients with steroid-sensitive nephrotic syndrome (SSNS) and steroid-resistant nephrotic syndrome (SRNS) in terms of CCL22 and Leptin levels.

**Methods:**

This prospective study included 117 children diagnosed with idiopathic nephrotic syndrome (INS). Peripheral blood samples were collected before initiating steroid therapy, and serum levels of CCL22 and Leptin were measured. Patients were categorized into three groups based on their response to steroid treatment. Renal biopsies were recommended for all children diagnosed with INS, with higher acceptance rates in glucocorticoid resistance patients.

**Results:**

Based on the response to steroid treatment, 117 children were divided as groups of SSNS (82 cases), frequent relapse nephrotic syndrome (FRNS) (10 cases), and SRNS (25 cases). A total of 41 patients underwent kidney biopsy, 11 cases (13.4%) in SSNS, 7 cases (70.0%) in FRNS and 24 cases (96.0%) in SRNS. 30 cases were minimal change disease (MCD), 9 cases were mesangial proliferative glomerulonephritis (MsPGN) and 3 cases were focal segmental glomerulosclerosis (FSGS). The levels of Leptin were significantly higher in SR patients (1208.1 ± 1044.1 pg/ml) compared to SS patients (515.4 ± 676.9 pg/ml) and controls (507.9 ± 479.8 pg/ml), regardless of the pathological type. CCL22 levels were significantly elevated in SRNS (92.2 ± 157.0 pg/ml), but the difference seemed to be attributed to the specific type of pathology, such as Minimal change disease (MCD) (127.4 ± 206.7 pg/ml) and focal segmental glomerulosclerosis (FSGS) (114.8 ± 22.0 pg/ml). For SRNS prediction, the AUC of Leptin, CCL22, and the joint prediction index were 0.764, 0.640, and 0.806, respectively.

**Conclusion:**

Serum levels of CCL22 and Leptin, detected prior to steroid therapy, were associated with steroid resistance in childhood INS.

## Introduction

Steroid-resistant nephrotic syndrome (SRNS) is characterized by the lack of response to steroid therapy, resulting in persistent proteinuria after four weeks of standard treatment (1). Among children diagnosed with nephrotic syndrome, approximately 10%–20% are clinically identified as having SRNS. Within five years of diagnosis, nearly 50% of children with SRNS progress to end-stage renal disease (2). The clinical manifestations of SRNS in children exhibit high heterogeneity, and its progression is primarily assessed through invasive pathological examination of kidney biopsies. The management of SRNS remains a significant challenge for pediatric nephrologists, and currently, there is no widely accepted tool for early prediction of steroid therapy resistance.

Studies have demonstrated an association between low steroid responsiveness and the regulation of T lymphocyte function. Patients with SRNS exhibit a decrease in T regulatory cells (Tregs), whereas an increase in Tregs has been observed in response to effective immunosuppressive or monoclonal antibody therapies (3). Tregs play a critical role in immunoregulation and exert immunosuppressive effects through various cellular and molecular mechanisms. They suppress CD4+ and CD8+ T cells, dendritic cells (DCs), B cells, natural killer (NK) cells, and macrophages (4).

Leptin and CCL22 have been identified as potential immunomodulators due to their interactions with Tregs. Leptin has been shown to sustain the activity of pro-inflammatory cytokines and immune cells, while also enhancing the immune response by stimulating M2 macrophages, promoting Th1 and Th17 cells, and inhibiting Tregs (5, 6). Inhibiting the Leptin pathway has been found to preserve Tregs proliferation and alleviate symptoms in certain autoimmune diseases (7). CCL22, a macrophage-derived immunosuppressive chemokine, acts through the CCL22-CCR4 axis to recruit Tregs. This occurs mainly in the secondary lymphoid organs and is eminently important for the control of adaptive immunity. Therefore, CCL22 most likely represents a central immune checkpoint that controls T-cell immunity (8).

In order to examine the involvement of Leptin and CCL22 in the development of SRNS, this study prospectively obtained serum samples from children with nephropathy prior to commencing steroid therapy. The patients were classified based on their response to steroid treatment, and the relationship between serum Leptin and CCL22 levels and steroid sensitivity in children with nephrotic syndrome was analyzed.

## Materials and methods

### Population

This study was conducted from January 2019 to September 2021 and involved children with idiopathic nephrotic syndrome who had not received prior steroid therapy. The study protocol involving human participants was reviewed and approved by the Ethics Committee.

The diagnosis of idiopathic nephrotic syndrome (INS) was based on the presence of edema, 24-h urinary protein excretion of ≥50 mg/kg, morning urinary protein/creatinine of >2 mg, hypoalbuminemia of <25 g/L and the disease of unknown causing. All children with INS received the standard steroid therapy and were classified into three categories, steroid-sensitive nephrotic syndrome (SSNS), frequent relapse nephrotic syndrome (FRNS) and steroid-resistant nephrotic syndrome (SRNS), on the basis of their clinical responses toward steroids. The SSNS group included patients with negative urinary protein for ≤4 weeks in those treated with sufficient prednisone [2 mg/(kg·d) or 60 mg/(m·d)]. The frequent relapse group included patients in whom INS recurred two times or more within half a year, or four times or more within 1 year in the course of the disease. The SRNS group included patients who failed to achieve remission after 4 weeks of daily sufficient prednisone. FRNS and SRNS were collectively referred to as refractory nephrotic syndrome (RNS). Further, the relapse group included patients in whom the quantity of urinary protein was ≥50 mg/kg, or the urinary protein/creatinine (mg/mg) of morning urine was ≥2.0, or the morning urinary protein changed from negative to positive for three consecutive days. The non-relapse group included patients in whom INS no recurred within 1 year after the first complete remission. The infrequent relapse group included patients in whom INS recurred once within 6 months or one to three times within 1 year after the first complete remission. Patients who were not finished four weeks of glucocorticoid therapy, or received other immunosuppressants, monoclonal antibodies, or cytotoxic drugs within four weeks, would be excluded from the study.

The clinical features of enrolled patients were recorded from the medical record, such as age, genders, creatinine, estimated glomerular filtration rate (eGFR), 24-h urine protein (Upro), occurred of complications and hypertension. eGFR was calculated according to Schwarz-formula (9).

### Sample collection

The blood sample were collected into vacuum sampling vessel containing coagulant (Improve Medical, China) before steroid therapy from the enrolled patients. Serum were separated within 2 h after blood collection and then divided and stored in the refrigerator at 2–8°C immediately. All the serum samples were transferred to an ultra-low temperature refrigerator (−80°C) no more than 8 h. The samples were guaranteed not to thaw until the ELISA test.

### Evaluation of renal biopsies

Renal biopsies were recommended in patients with INS diagnosis. Pathology test should obtain the written informed consent of the guardian first. Renal biopsies were executed by skilled nephrologists, and all biopsies were assessed by pathologists through light microscopy and immunofluorescence. Each kidney sample was observed for the total number of glomeruli, glomerular sclerosis, mesangial proliferation, basement membrane thickening, tubular degeneration and atrophy, interstitial fibrosis, interstitial inflammation, etc.

### ELISA tests

Human Leptin ELISA KIT (4A biotech, China, Assay range: 31.25–2000 pg/ml), Human CCL22 ELISA KIT (4A biotech, China, Assay range: 15.625–1000 pg/ml) were used for patient serum test. Briefly, to assay each protein, serum samples at an optimal dilution were added to a microplate precoated with capture antibody, incubated, washed and followed by addition of capture antibody, horseradish peroxidase and substrate. The absolute levels of serum protein biomarkers were determined using standard curves run on each ELISA plate, and normalized by urine creatinine concentration.

### Statistical analysis

The quantitative data with normal distribution was expressed as (Average ± SD), and the classified data indicated with the quantity of each component separately. Statistical analysis was performed using SPSS 22.0. The chi-square test was used to analyze gender differences among the groups, while the *t*-test was used to assess age differences. Outlier detected by histogram, and replaced with the average in statistical process. Pearson bivariate correlation analysis was used for correlation statistics. A *P*-value greater than 0.05 indicated no significant difference. After quantifying the ELISA results based on the standard curve, the *t*-test or Wilcoxon rank sum test was used for between-group comparisons, and analysis of variance or Kruskal-Wallis test was used for comparisons involving more than two groups. Statistical significance was set at *P* < 0.05. Receiver operating characteristic (ROC) curves were utilized to determine the effective area, sensitivity, and specificity of candidate indexes.

## Results

### Clinical features

A total of 117 cases (86 males, 31 females) with childhood INS were included in the study, comprising the subgroups of SSNS (82 cases), FRNS (10 cases), and SRNS (25 cases). Additionally, 40 cases (28 males, 12 females) undergoing health check-ups and confirmed to be free of obvious diseases were selected as the healthy control group. Serum samples were collected before steroid therapy and stored according to the aforementioned protocol. The clinical features of children enrolled in the study were listed in Table 1. There were no significant differences in age, gender ratio, Creatinine, eGFR and Upro between the three groups (*P *> 0.05). There were 148 complications could be recorded in 117 INS patients, with 12 types. The first three kinds were inflammation (48 cases), hydrops (30 cases) and liver injury (23 cases). There was no difference in the incidence of complications among three subgroups (*P *> 0.05). The incidence of hypertension was different (*P *< 0.05), with the highest in SRNS.

**Table 1:** The clinical features of children enrolled in the study.

Clinical features	INS				Healthy	*P*-value
	Total	SSNS	FRNS	SRNS		
Cases, *n*	117	82	10	25	40	/
Age, months	48.5 ± 36.4	46.1 ± 33.3	45.3 ± 23.2	57.4 ± 47.5	66.1 ± 17.8	0.389
Genders, male/female	86/31	63/19	7/3	16/9	28/12	0.607
Creatinine, μmol/L	39.9 ± 13.9	39.4 ± 13.7	39.9 ± 3.99	41.2 ± 16.6	/	0.860
eGFR, ml/min/1.73 m^2^	130.0 ± 42.9	132.4 ± 45.6	119.2 ± 20.4	126.5 ± 39.4	/	0.600
24-h urine protein, mg/24 h	2526.7 ± 2803.8	2225.2 ± 2242.8	2219.6 ± 1277.4	3797.9 ± 4282.9	/	0.065
Complications, numbers/per capite	148/1.26	104/1.27	10/1.00	34/1.36	/	0.838
Hypertension, *n* (%)	8 (6.8%)	2 (2.4%)	1 (10.0%)	5 (20.0%)	/	**0** **.** **009**

SSNS, steroid-sensitive nephrotic syndrome; FRNS, frequent relapse nephrotic syndrome; SRNS, steroid-resistant nephrotic syndrome, *P*-value: <0.05 means statistically significant difference; eGFR, estimated glomerular filtration rate.

The bold values mean *P* < 0.05 with the significant difference.

A total of 41 (35.0%) patients underwent kidney biopsy, 11 cases (13.4%) in SSNS, 7 cases (70.0%) in FRNS and 24 cases in SRNS with an acceptance rate up to 96.0%. Minimal change disease (MCD) was the most common pathological type with 30 cases, mesangial proliferative glomerulonephritis (MsPGN) with 9 cases and focal segmental glomerulosclerosis (FSGS) with 3 cases. Pathological types in the three groups with serum concentration of CCL22 and Leptin were shown in Table 2.

**Table 2:** Pathological types in three groups with serum concentration of CCL22 and Leptin.

Groups	Pathological types	Cases	Leptin (pg/ml)	*P*-value to MsPGN	CCL22 (pg/ml)	*P*-value to MsPGN
SSNS						
	MsPGN	1	377.8	/	24.2	/
	MCD	10	1139.1 ± 1221.1	/	36.6 ± 36.4	/
FRNS						
	MCD	7	1201.7 ± 1247.6	/	17.7 ± 5.4	/
SRNS						
	MsPGN	8	1320.9 ± 1189.8	/	26.6 ± 15.3	/
	MCD	13	1206.5 ± 1067.1	0.822	73.2 ± 70.2	0.189
	FSGS	3	914.6 ± 753.9	0.601	114.8 ± 22.0	**<0**.**001**
Total		42	1200.2 ± 1089.4	/	67.2 ± 122.7	/
	MsPGN	9	1216.1 ± 1156.5	**/**	26.3 ± 14.4	**/**
	MCD	30	1253.0 ± 1204.8	0.984	49.9 ± 55.4	0.086
	FSGS	3	914.6 ± 753.9	0.624	114.8 ± 22.0	**<0**.**001**

SSNS, steroid-sensitive nephrotic syndrome; FRNS, frequent relapse nephrotic syndrome; SRNS, steroid-resistant nephrotic syndrome; MCD, minimal change disease; MsPGN, mesangial proliferative glomerulonephritis; FSGS, focal segmental glomerular sclerosis; *P*-value: <0.05 means statistically significant difference.

The bold values mean *P* < 0.05 with the significant difference.

### Leptin and CCL22 had no significant correlation with clinical features

There was no significant correlation between Leptin and Creatinine (*P* = 0.368), eGFR (*P* = 0.329), Upro (*P* = 0.241). At the same between CCL22 and Creatinine (*P* = 0.060), eGFR (*P* = 0.570), Upro (*P* = 0.963). These indicated that changes of Leptin and CCL22 in INS patients had a different significance compared to clinical features.

### Leptin levels were elevated in RNS, especially in cases of SRNS

**Figure 1:**
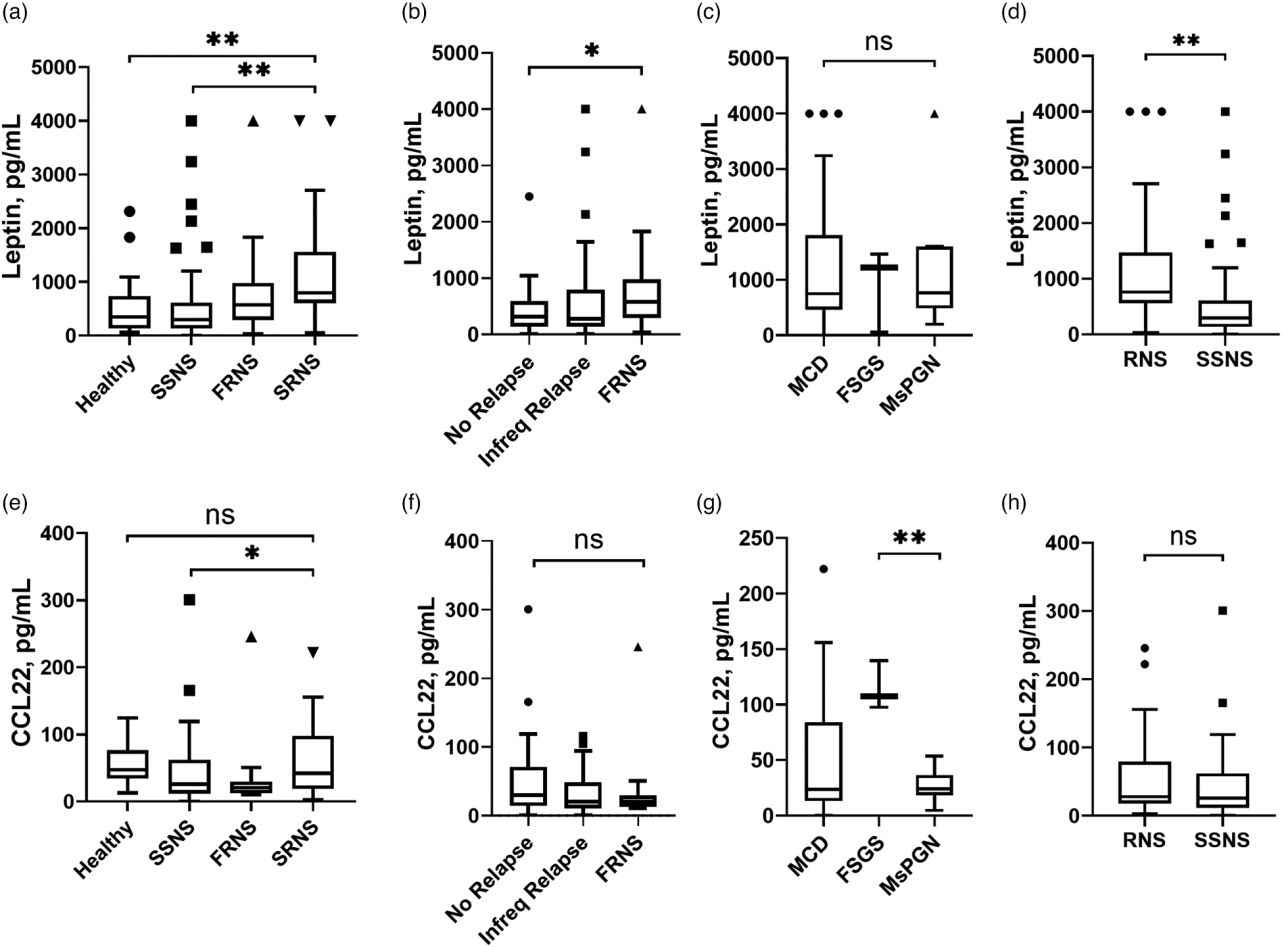
Serum concentration of Leptin and CCL22 in INS patients compared between different subgroups. INS, idiopathic nephrotic syndrome; SSNS, steroid-sensitive nephrotic syndrome; FRNS, frequent relapse nephrotic syndrome; SRNS, steroid-resistant nephrotic syndrome; RNS, refractory nephrotic syndrome; No Relapse, no recurred within 1 year after the first complete remission; Infreq Relapse, recurred once within 6 months or one to three times within 1 year after the first complete remission; MCD, minimal change disease; MsPGN, mesangial proliferative glomerulonephritis; FSGS, focal segmental glomerular sclerosis; *, statistically significant difference with *P* < 0.05; **, statistically significant difference with *P* < 0.01; ns, non-significant.

The concentration of serum Leptin was significantly increased in RNS, with even higher levels observed in the SRNS subgroup. No significant differences were found between the healthy group and SSNS. The results were depicted in Figure 1A. Patients in SSNS were distinguished as no relapse and infrequent relapse with fewer recurrences than FRNS. Leptin was associated with the frequency of relapse, and patients with FRNS had significantly elevated Leptin concentrations (Figure 1B). Regarding the different pathological types, serum Leptin concentration was increased compared to healthy children, with a significant increase observed in MCD and MsPGN (Figure 1C). In patients with steroid resistance, there were minimal differences in Leptin concentration among the three pathological types (Table 2). Compared with group SSNS, Leptin was significantly higher in RNS group (Figure 1D). All data were listed in Table 2 and Table 3.

**Table 3:** Serum concentration of CCL22 and Leptin in INS patients with different steroid sensitivity.

Group	Cases	Leptin (pg/ml)	*P*-value to healthy	CCL22 (pg/ml)	*P*-value to healthy
Healthy	40	507.9 ± 479.8	/	55.9 ± 28.3	/
INS	117	697.2 ± 852.9	0.185	51.3 ± 84.2	0.737
SSNS	82	515.4 ± 676.9	0.950	40.6 ± 44.2	**0**.**048**
No relapse	42	406.5 ± 419.6	0.311	46.6 ± 53.2	0.333
Infreq relapse	40	629.8 ± 860.4	0.436	34.2 ± 31.6	**0**.**002**
RNS	35	1123.1 ± 1060.2	**0**.**001**	55.8 ± 62.0	0.994
FRNS	11	937.8 ± 1122.2	0.063	42.0 ± 68.5	0.312
SRNS	24	1208.1 ± 1044.1	**0**.**001**	62.4 ± 59.0	0.556

INS, idiopathic nephrotic syndrome; SSNS, steroid-sensitive nephrotic syndrome; FRNS, frequent relapse nephrotic syndrome; SRNS, steroid-resistant nephrotic syndrome; RNS, refractory nephrotic syndrome; No Relapse, no recurred within 1 year after the first complete remission; Infreq Relapse, recurred once within 6 months or one to three times within 1 year after the first complete remission; *P*-value: <0.05 means statistically significant difference.

The bold values mean *P* < 0.05 with the significant difference.

### CCL22 decreased in SSNS

The concentration of serum CCL22 was significantly decreased in SSNS compared with SRNS, and no significant differences with healthy group (Figure 1E). The recurrence of INS patients was not related to CCL22 concentration (Figure 1F). CCL22 differed significantly among the three pathological types, with elevated levels in MCD and FSGS, and reduced levels in MsPGN (Figure 1G). Compared between RNS and SSNS, there were no significant differences in CCL22 concentration (Figure 1H). All data were listed in Table 2 and Table 3.

### CCL22 and Leptin levels were elevated in INS patients with steroid resistance

**Figure 2:**
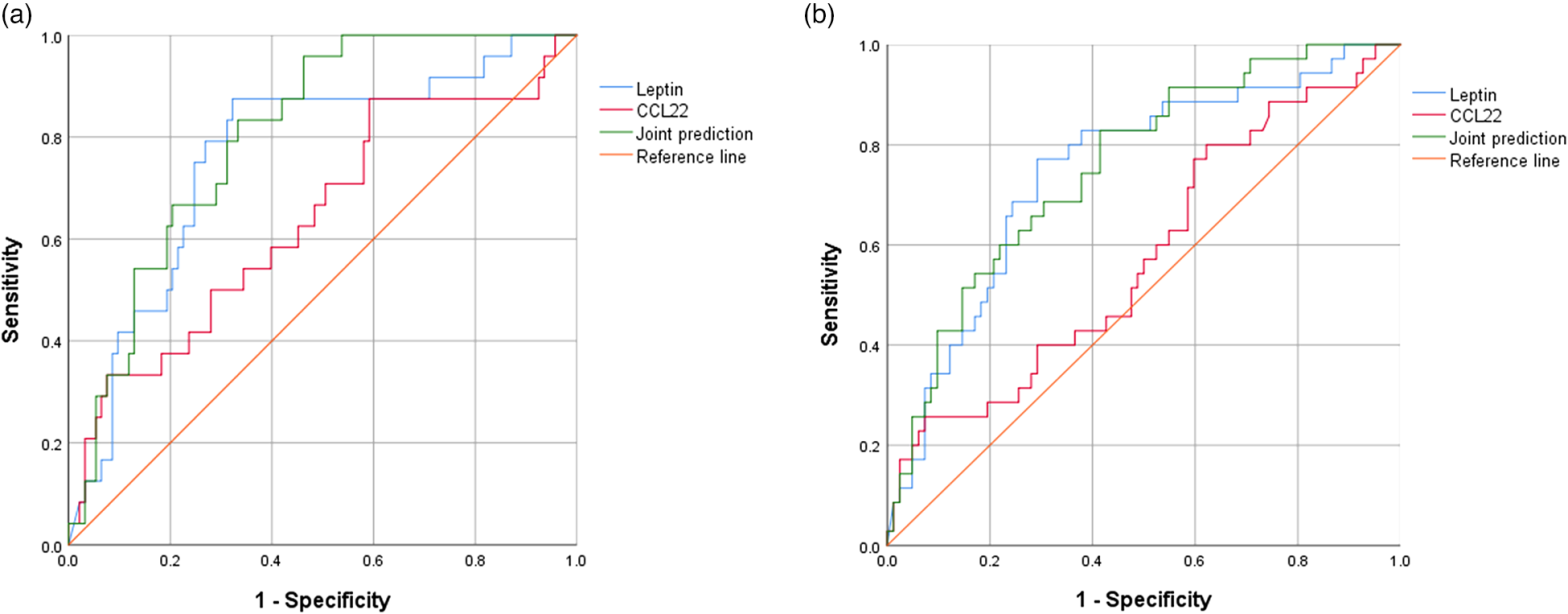
ROC curves of serum CCL22 and Leptin to predict SRNS or RNS. (**A**) For SRNS prediction. (**B**) For RNS prediction. Joint prediction: Joint diagnostic performance prediction by CCL22 and Leptin.

Compared to SSNS group, the concentration of serum CCL22 and Leptin significantly increased in SRNS (*P *= 0.046 and *P *= 0.007). The diagnostic performance of serum CCL22 and Leptin in predicting RNS or SRNS was evaluated using the ROC curve shown in Figure 2. For SRNS prediction, the AUC of Leptin, CCL22, and the joint prediction index were 0.764, 0.640, and 0.806, respectively (Figure 2A). The cut-off of Leptin was 602.6 pg/ml, with positive predictive value (PPV) = 0.43, negative predictive value (NPV) = 0.93, The cut-off of CCL22 was 23.7 pg/ml, with PPV = 0.27, NPV = 0.87. For RNS prediction, the AUC of Leptin, CCL22, and the joint prediction index were 0.748, 0.578, and 0.756, respectively (Figure 2B). The cut-off of Leptin was 555.6 pg/ml, with PPV = 0.53, NPV = 0.88, The cut-off of CCL22 was 18.3 pg/ml, with PPV = 0.36, NPV = 0.80.

## Discussion

The present study focused on the serum concentrations of CCL22 and Leptin in patients with idiopathic nephrotic syndrome. By prospectively collecting samples and conducting pre-steroid tests, we observed differences in CCL22 and Leptin concentrations among groups of patients with varying steroid responses. This study demonstrated the potential of CCL22 and Leptin to predict steroid resistance in the early stages of nephrotic syndrome.

Tregs play a crucial role in immune tolerance and are recognized as regulators of inflammation in INS. Previous research has shown that patients with steroid-resistant nephrotic syndrome exhibit lower levels of Tregs compared to those with steroid sensitivity (10). A slower increase in Tregs counts from disease onset to remission has been associated with a higher frequency of INS relapses (11). Severe disorders in lymphocyte subsets and abnormal regulation are believed to be involved in SRNS (12). Lymphocyte subsets, especially Tregs, may influence the treatment and prognosis of corticosteroids in INS.

Measuring Leptin level is a hot topic now, and more topics are focusing on its measurement as early childhood obesity, early childhood developmental assessment scores (13, 14). Our study provides confirmation that elevated Leptin levels are associated with steroid resistance in INS. It can be hypothesized that increased Leptin may diminish the response of kidney disease to steroid therapy by suppressing Treg levels, which aligns with findings from other studies in SRNS patients. The relationship between Leptin and steroid sensitivity has also been reported. Henmi K reported a significant decrease in PBMC response to prednisolone in INS patients and postulated that the Leptin receptor (OB-R) plays a crucial role (15). Increased serum Leptin has also been identified as a negative prognostic factor for the response to steroid therapy in autoimmune hepatitis (16).

CCL22 is an important factor in facilitating the migration of Tregs both *in vitro* and *in vivo* (17). In our study, there were significant differences between SRNS and SSNS patients in CCL22 concentrations, and were observed among patients with different pathological types. CCL22 levels were significantly elevated in the MCD and FSGS pathological types. We can infer that CCL22 may be related to the pathological changes in the glomerular structure. But more research data was needed to prove this.

As a small-sample, single-center observational study, our research has certain limitations. Expanding the sample size and conducting multi-center studies would provide more compelling conclusions. Furthermore, further investigations into the mechanisms of other related molecules would enhance the credibility of our findings and pave the way for subsequent explorations of the underlying mechanisms.

In conclusion, this study has demonstrated an association between serum concentrations of CCL22 and Leptin, measured prior to steroid therapy, and steroid resistance in childhood idiopathic nephrotic syndrome. Additionally, there were notable variations in CCL22 concentration across different glomerular pathological types, highlighting the need for further investigation into the molecular mechanisms involved.

## Data Availability

The datasets presented in this study can be found in online repositories. The names of the repository/repositories and accession number(s) can be found below: https://figshare.com/, 10.6084/m9.figshare.23704074.
